# Supplementary material for: Content-rich biological network constructed by mining PubMed abstracts
Source: BMC Bioinformatics. 2004 Oct 8;5:147. doi: 10.1186/1471-2105-5-147 (PMC528731; doi:10.1186/1471-2105-5-147)
Supplement: Additional File 2 — The original results of the above study (non-essential files are deleted to keep the file size under the limit set by BMC bioinformatics). [file 1471-2105-5-147-S2.bz2 › chilibotAdditionalFile2/dip05/317ID10797014E2428/html/UL18_UL38.html]

 


 **UL18** and **UL38** 
  
Found 8 abstracts in PubMed, retrieved 05.  
 

 What does Google say? 
 PDF only 
| .edu only 

---

**Interactive relationship** (e.g. stimulation, inhibition, etc)

**Non-interactive relationship** (e.g. studied together, co-existance, homology, etc.)

- coded by the UL19 gene and two minor proteins, VP19C  **UL38**  gene and VP23  **UL18**  gene.  Ref: 8918599 J Mol Biol, 1996
- Herpes simplex virus type 1 HSV 1 intermediate capsids are composed of seven proteins, VP5, VP19C, VP21, VP22a, VP23, VP24, and VP26, and the genes that encode these proteins, UL19,  **UL38** , UL26, UL26.5,  **UL18** , UL26, and UL35, respectively.  Ref: 7745718 J Virol, 1995
- The proteins coded by the five major capsid genes of herpes simplex virus 1, VP5 gene UL19, VP19c  **UL38** , VP23  **UL18** , pre VP22a UL26.5, and pre VP21 UL26, assemble into fragile roundish procapsids, which mature into robust polyhedral capsids in a transition similar to that undergone by bacteriophage proheads.  Ref: 8918600 J Mol Biol, 1996
- The proteins encoded by  **UL18**  VP23, UL19 VP5, UL35 VP26 and  **UL38**  VP19C are components of the outer capsid shell whereas those specified by UL26 VP21 and VP24 and UL26.5 VP22a, are involved in scaffold formation.  Ref: 8176371 J Gen Virol, 1994
